# Clinical characteristics and survival in early-onset lung cancer: a multicenter cohort study

**DOI:** 10.3389/fonc.2026.1906257

**Published:** 2026-08-12

**Authors:** Hongliang Liu, Bibo Li, Zhikai Yu, Jia Du, Xiu Liu, Yan Zhang, Qing Guo, Lihong Mu, Mei He

**Affiliations:** 1 Department of Epidemiology, School of Public Health, Research Center for Medicine and Social Development, Chongqing Medical University, Chongqing, China; 2 Department of Oncology, Chongqing General Hospital & Chongqing Academy of Medical Sciences, Chongqing, China; 3 Office of Cancer Prevention and Control, Chongqing University Cancer Hospital, Chongqing, China

**Keywords:** clinical characteristic, early-onset lung cancer, molecular, prognosis, survival

## Abstract

**Introduction:**

The characteristics of early-onset lung cancer (EOLC) have not been extensively studied. Our research aimed to comprehensively assess the clinicopathological, genetic features and prognosis of EOLC.

**Materials and methods:**

In this retrospective study of lung cancer patients diagnosed at 26 Chongqing hospitals between January 2019 and December 2022 (18-74 years), we stratified them into early-onset lung cancer (<50 years) and late-onset lung cancer (≥50 years) groups. Furthermore, we used propensity score matching to balance baseline characteristics, compare overall survival and lung cancer-specific survival between early-onset lung cancer (EOLC) and late-onset lung cancer (LOLC) groups, and perform subgroup comparisons.

**Results:**

A total of 6,883 lung cancer patients were included in the final analysis, comprising 690 patients with EOLC and 6,193 patients with LOLC. Compared with the LOLC group, the EOLC group had more females and never-smokers. In clinical features, EOLC patients predominantly had adenocarcinoma (69.7% vs. 52.2%), more often presented with stage IV disease (49.3% vs. 44.0%), and had higher rates of bone (22.8% vs. 16.9%) and brain metastases (18.4% vs. 10.8%), yet were more likely to undergo surgery (31.0% vs. 22.4%). Significant differences in mutation rates were observed for HER2 (10.1% vs. 1.6%). After propensity score matching, late-onset was identified as an independent risk factor for adverse survival outcomes, the median overall survival was 42.7 months for EOLC versus 32.9 months for LOLC, with 1-, 3-, and 5-year survival rates of 78.0%, 54.4%, and 42.2% in the EOLC group, compared with 75.5%, 47.8%, and 35.4% in the LOLC group.

**Conclusions:**

Despite more aggressive clinicopathological features, EOLC was associated with better survival outcomes. Moreover, distinct driver gene alteration profiles highlight the need to identify targetable alterations and implement targeted therapy in this enriched population.

## 1 Introduction

Lung cancer was the leading cause of cancer incidence and mortality in 2022, with almost 2.5 million new cases and over 1.8 million deaths worldwide (1). In recent decades, lung cancer incidence has declined in many high-income countries, owing to the implementation of screening programs and effective tobacco control policies, most notably the sustained reduction in smoking prevalence (2–4). However, the incidence of early-onset lung cancer (EOLC) in adults younger than 50 years is clearly increasing globally (5). Asia accounted for 75.9% of global EOLC cases, with China contributing 64.1% of these cases (6). The underlying causes of the rising incidence of early-onset lung cancer remain poorly understood but may be related to changes in environmental and lifestyle factors, as well as the widespread use of CT scans in routine check-up programs (7, 8).

Lung cancer in younger patients has been far less studied than late-onset lung cancer (LOLC) in adults aged ≥50 years. Moreover, reports on differences in tumor biology, clinical presentation, and prognosis between early-onset and late-onset lung cancer patients remain conflicting. Some studies have observed that early-onset lung cancer may exhibit greater aggressiveness, yet better survival compared to late-onset lung cancer (9, 10). In contrast, other studies report that lung cancer in younger patients may have a significantly poorer prognosis than that in older patients (11, 12). Furthermore, the contributions of genetic factors or unique mutagenic processes to early-onset lung cancer remain poorly understood.

To address this gap, we performed a multicenter cohort study that systematically characterized the clinical presentation, genetic mutations, and prognosis of patients with early-onset lung cancer in Southwest China. Our findings offer a comprehensive understanding of this disease in young East Asian patients and provide a scientific basis for personalized screening and intervention strategies.

## 2 Materials and methods

### 2.1 Patient selection

**Figure 1:**
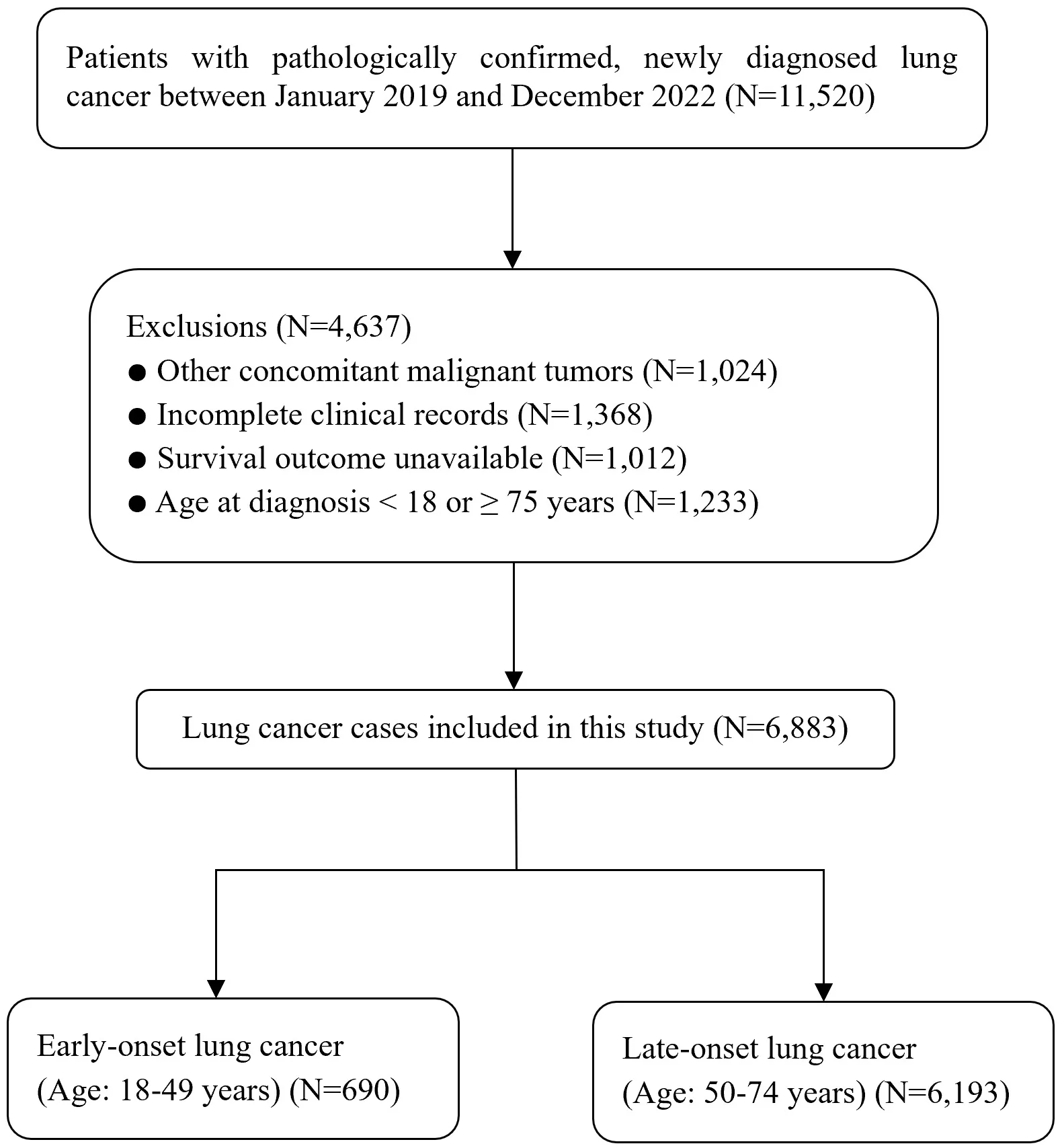
Flowchart of patient selection and classification in the study cohort.

We conducted a retrospective review of 11,520 consecutive patients with pathologically confirmed lung cancer, diagnosed between January 2019 and December 2022 at 26 Grade II or higher hospitals offering oncology diagnosis and treatment services in Chongqing. Of these patients, exclusions included 1,024 with other concomitant malignant tumors, 1,368 with incomplete clinical information, 1,012 with unavailable survival outcomes, and 1,233 with an age of <18 or >75 years (**Figure 1**). A total of 6,883 lung cancer patients were ultimately included. Guidelines for the diagnosis and treatment of common cancers from the Chinese Anti-Cancer Association and the National Cancer Research and Cancer Office were followed. The protocols for data collection and analysis were approved by the Institutional Review Board of Chongqing University Cancer Hospital. The requirement for informed consent from patients was waived due to the retrospective nature of the study. Confidentiality of patient data was maintained throughout the study.

### 2.2 Grouping criteria

We defined early-onset lung cancer as occurring in individuals aged < 50 years for the following reasons. First, this cutoff is among the most frequently adopted definitions in large epidemiological and molecular studies, ensuring both consistency with previous research and comparability across studies (13–15). Second, compared with older patients, the population below this age threshold generally maintains relatively intact physiological function and has less cumulative exposure to age-related comorbidities (16, 17). Third, and more importantly, our findings empirically support the use of this 50-year cutoff, as evidenced by restricted cubic spline (RCS) and piecewise Cox regression analyses in our cohort (**Supplementary Material Table 1**; **Figure 1**).

### 2.3 Data collection

We collected the following information for each patient: 1) demographic characteristics, including sex, age at diagnosis, marital status, smokers (never, former, current), and family history of lung cancer. Current smokers were defined as individuals who had smoked ≥ 100 cigarettes (or equivalent tobacco) in their lifetime and were still smoking at diagnosis. Former smokers had smoked ≥100 cigarettes (or equivalent) in their lifetime but had quit at least 6 months before diagnosis. Never smokers were defined as those who had never smoked daily or smoked < 100 cigarettes (or equivalent) in their lifetime (18). Patients were considered to have a significant family history if they had either one first-degree relative or at least two second-degree relatives diagnosed with lung cancer. 2) Clinical characteristics including tumor site(left, right, bilateral), tumor staging(0–I, II, III, IV), tumor size (≤3, 3.01–5, >5 cm), pleural invasion, bone metastasis, brain metastasis, liver metastasis, histology(adenocarcinoma, squamous cell carcinoma, small cell lung cancer, others), treatment (surgery, radiotherapy, chemotherapy, targeted therapy, immunotherapy, supportive care, untreated). Tumor staging was evaluated according to the 9th edition of the American Joint Committee on Cancer (AJCC) staging system. The histological subtypes of lung cancer were defined according to the International Classification System (19). Lung cancer cases were sub-divided into adenocarcinoma (ADC) (M8140, M8144, M8211, M8230, M8250, M8251, M8255, M8260, M8333, M8549, M8550, M8574, or M8576), squamous cell carcinoma (SCC) (M8070, M8083, M8072, M8074, or M8076), small cell carcinoma (SCC) (M8041, or M8045), others including large cell carcinoma (LCC) (M8012, M8013, M8246, or M8310), adeno-squamous carcinoma (ASC)(M8560). Systemic therapy encompasses chemotherapy, targeted therapy, and immunotherapy. 3) The death outcomes of lung cancer patients were ascertained through the Chongqing Resident Cause of Death Surveillance System, which covers all deaths in the entire population of Chongqing, using unique ID numbers to link records. The study endpoints were overall survival (OS) and lung cancer-specific survival (CSS). The outcome event was death, and the follow-up cutoff date was December 31, 2025. Overall survival was defined as the time from diagnosis to death from any cause, and lung cancer-specific survival was defined as the time from diagnosis to death from lung cancer. Patients who had not experienced the outcome event by the end of the observation period were defined as censored.

### 2.4 Molecular characteristics

Molecular characteristics were assessed using a validated real-time fluorescent quantitative amplification refractory mutation system polymerase chain reaction (ARMS-PCR) assay (20). The analyzed driver genes included EGFR mutations in the kinase domain, ALK rearrangements, ROS1 rearrangements, HER2 mutations in the kinase domain, BRAF mutations, RET rearrangements, KRAS mutations, and MET exon 14 skipping mutations. Molecular data were retrospectively extracted from the Chongqing Cancer Registry System database. All data in this study were derived from routine patient care, and not all patients underwent molecular testing. Gene-specific analyses therefore included only those with available testing records. Patients without such records were treated as having missing data and excluded from the corresponding analyses. Additionally, due to financial constraints, some patients underwent testing for only one or a few targets, resulting in variable sample sizes across individual driver genes. Consequently, the molecular analyses in this study were prespecified as exploratory.

### 2.5 Propensity score matching

To minimize baseline differences between patients with EOLC and LOLC, propensity score matching (PSM) was performed using the “MatchIt” package in R. Propensity scores were estimated using a multivariable logistic regression model with onset type (EOLC vs. LOLC) as the dependent variable. Covariates included sex, smokers, family history, tumor staging, tumor size, histology, bone metastasis, brain metastasis, and treatment. Patients were matched using nearest-neighbor matching without replacement at a 1:3 ratio with a caliper width of 0.05. Covariate balance before and after matching was evaluated using absolute standardized mean differences (SMDs), with an absolute SMD <0.10 indicating adequate balance. Covariate balance is presented in the **Supplementary Material Figure 2**.

### 2.6 Statistical analysis

All statistical analyses were performed using R 4.4.3 software (R Foundation for Statistical Computing). Continuous variables were expressed as mean ± standard deviation (SD). Categorical variables were summarized as frequencies and percentages. Using the chi-square test to compare differences between groups. OS and CSS were estimated using the Kaplan–Meier method and compared using the log-rank test. Cox proportional hazards regression models were applied to estimate hazard ratios (HRs) and 95% confidence intervals (CIs). The proportional hazards assumption was assessed using Schoenfeld residuals. Missing data were handled using complete-case analysis. Subgroup analyses were performed using Cox regression. All statistical tests were two-sided, and P < 0.05 was considered statistically significant.

## 3 Results

### 3.1 Demographic and clinical characteristics

The demographic and clinical characteristics of 690 EOLC cases and 6193 LOLC cases are shown in **Table 1**. The mean age was 44.1 ± 5.5 years in the EOLC group and 63.2 ± 6.8 years in the LOLC group. In the EOLC cohort, 54.3% were male, corresponding to a male-to-female ratio of 1.2:1. Compared with patients with LOLC, those with EOLC were more likely to be female (45.7% vs. 27.6%, SMD = 0.382) and never-smokers (65.8% vs. 47.1%, SMD = 0.387). Clinically, EOLC patients presented with more advanced disease, including a higher proportion of stage IV cases (49.3% vs. 44.0%, SMD = 0.125). In addition, adenocarcinoma was more common in EOLC (69.7% vs 52.2%, SMD = 0.419), with higher frequencies of bone metastasis (22.8% vs 16.9%, SMD = 0.146) and brain metastasis (18.4% vs 10.8%, SMD = 0.218). Surgery was also more frequently performed in the EOLC group than the LOLC group (31.0% vs 22.4%, SMD = 0.336). After PSM, 686 EOLC patients were successfully matched to 2,027 LOLC patients. No significant differences in baseline characteristics were observed between the matched groups (all SMD < 0.1). The Love plot showed that the SMDs for all covariates were <0.1 after matching, indicating good balance (**Supplementary Material Figure 2**).

**Table 1:** Baseline demographic and clinicopathological characteristics of EOLC and LOLC patients.

Varibales	Before PSM		SMD	After PSM		SMD
	EOLC(n=690,%)	LOLC(n=6193,%)		EOLC(n=686,%)	LOLC(n=2027,%)	
Mean age	44.1 ± 5.5	63.2 ± 6.8		44.1 ± 5.5	62.6 ± 6.9	
Sex			0.382			0.018
Male	375(54.3)	4486(72.4)		374(54.5)	1087(53.6)	
Female	315(45.7)	1707(27.6)		312(45.5)	940(46.4)	
Marital status			0.009			0.018
Married	647(93.8)	5793(93.5)		643(93.7)	1891(93.3)	
Unmarried/Divorced/Widowed	43(6.2)	400(6.5)		43(6.3)	136(6.7)	
Ethnicity			0.064			0.021
Han	669(97.0)	6067(98.0)		665(96.9)	1972(97.3)	
Others	21(3.0)	126(2.0)		21(3.1)	55(2.7)	
Smokers			0.387			0.029
Never smokers	454(65.8)	2914(47.1)		450(65.6)	1332(65.7)	
Former smokers	70(10.1)	1067(17.2)		70(10.2)	222(11.0)	
Current smokers	166(24.1)	2212(35.7)		166(24.2)	473(23.3)	
Family history			0.080			0.048
Yes	44(6.4)	282(4.6)		42(6.1)	102(5.0)	
No	646(93.6)	5911(95.4)		644(93.9)	1925(95.0)	
Tumor site			0.028			0.052
Left	301(43.6)	2758(44.5)		298(43.4)	929(45.8)	
Right	368(53.3)	3271(52.8)		367(53.5)	1045(51.6)	
Bilateral	21(3.0)	164(2.6)		21(3.1)	53(2.6)	
Tumor staging			0.125			0.031
0-I	201(29.1)	1845(29.8)		201(29.3)	615(30.3)	
II	42(6.1)	430(6.9)		42(6.1)	114(5.6)	
III	107(15.5)	1194(19.3)		107(15.6)	321(15.8)	
IV	340(49.3)	2724(44.0)		336(49.0)	977(48.2)	
Tumor size, cm			0.173			0.027
≤3	444(64.3)	3542(57.2)		441(64.3)	1297(64.0)	
3.01-5	158(22.9)	1512(24.4)		157(22.9)	483(23.8)	
>5	88(12.8)	1139(18.4)		88(12.8)	247(12.2)	
Pleural invasion			0.083			0.063
Yes	166(24.1)	1276(20.6)		166(24.2)	437(21.6)	
No	524(75.9)	4917(79.4)		520(75.8)	1590(78.4)	
Bone metastasis			0.146			0.024
Yes	157(22.8)	1049(16.9)		155(22.6)	438(21.6)	
No	533(77.2)	5144(83.1)		531(77.4)	1589(78.4)	
Brain metastasis			0.218			0.074
Yes	127(18.4)	667(10.8)		123(17.9)	308(15.2)	
No	563(81.6)	5526(89.2)		563(82.1)	1719(84.8)	
Liver metastasis			0.005			0.004
Yes	46(6.7)	421(6.8)		45(6.6)	135(6.7)	
No	644(93.3)	5772(93.2)		641(93.4)	1892(93.3)	
Histology			0.419			0.038
Adenocarcinoma	481(69.7)	3230(52.2)		477(69.5)	1418(70.0)	
Squamous cell carcinoma	102(14.8)	1928(31.1)		102(14.9)	306(15.1)	
Small cell carcinoma	82(11.9)	774(12.5)		82(12.0)	243(12.0)	
Others	25(3.6)	261(4.2)		25(3.6)	60(3.0)	
Treatment			0.336			0.030
Surgery	214(31.0)	1389(22.4)		213(31.0)	626(30.9)	
Radiotherapy	31(4.5)	347(5.6)		31(4.5)	91(4.5)	
Chemotherapy	169(24.5)	1210(19.5)		166(24.2)	510(25.2)	
Targeted therapy	83(12.0)	650(10.5)		83(12.1)	238(11.7)	
Immunotherapy	30(4.3)	595(9.6)		30(4.4)	92(4.5)	
Supportive care	116(16.8)	1428(23.1)		116(16.9)	328(16.2)	
Untreated	47(6.8)	574(9.3)		47(6.9)	142(7.0)	

EOLC, early-onset lung cancer; LOLC, late-onset lung cancer; PSM, propensity score matching; SMD, standardized mean difference.

Given the higher proportion of stage IV among EOLC patients, treatment patterns were further explored in this subgroup (**Table 2**). Significant differences in treatment distribution were observed between EOLC and LOLC patients (P = 0.005). Compared with LOLC patients, EOLC patients more frequently underwent chemotherapy (26.8% vs. 21.2%) and targeted therapy (21.2% vs. 16.4%), whereas immunotherapy (5.9% vs. 10.7%) and supportive care (17.6% vs. 20.9%) were less frequently administered. The proportion of untreated patients was comparable between the two groups (6.8% vs. 7.6%).

**Table 2:** Distribution of first-line treatment strategies among patients with stage IV.

Treatment	EOLC (n=340, %)	LOLC (n=2724, %)	P
Treatment for IV patients			0.005
Surgery	52(15.3)	399(14.6)	
Radiotherapy	22(6.5)	233(8.6)	
Chemotherapy	91(26.8)	578(21.2)	
Targeted therapy	72(21.2)	446(16.4)	
Immunotherapy	20(5.9)	291(10.7)	
Supportive care	60(17.6)	570(20.9)	
Untreated	23(6.8)	207(7.6)	

EOLC, early-onset lung cancer; LOLC, late-onset lung cancer.

### 3.2 Survival analysis

**Figure 2:**
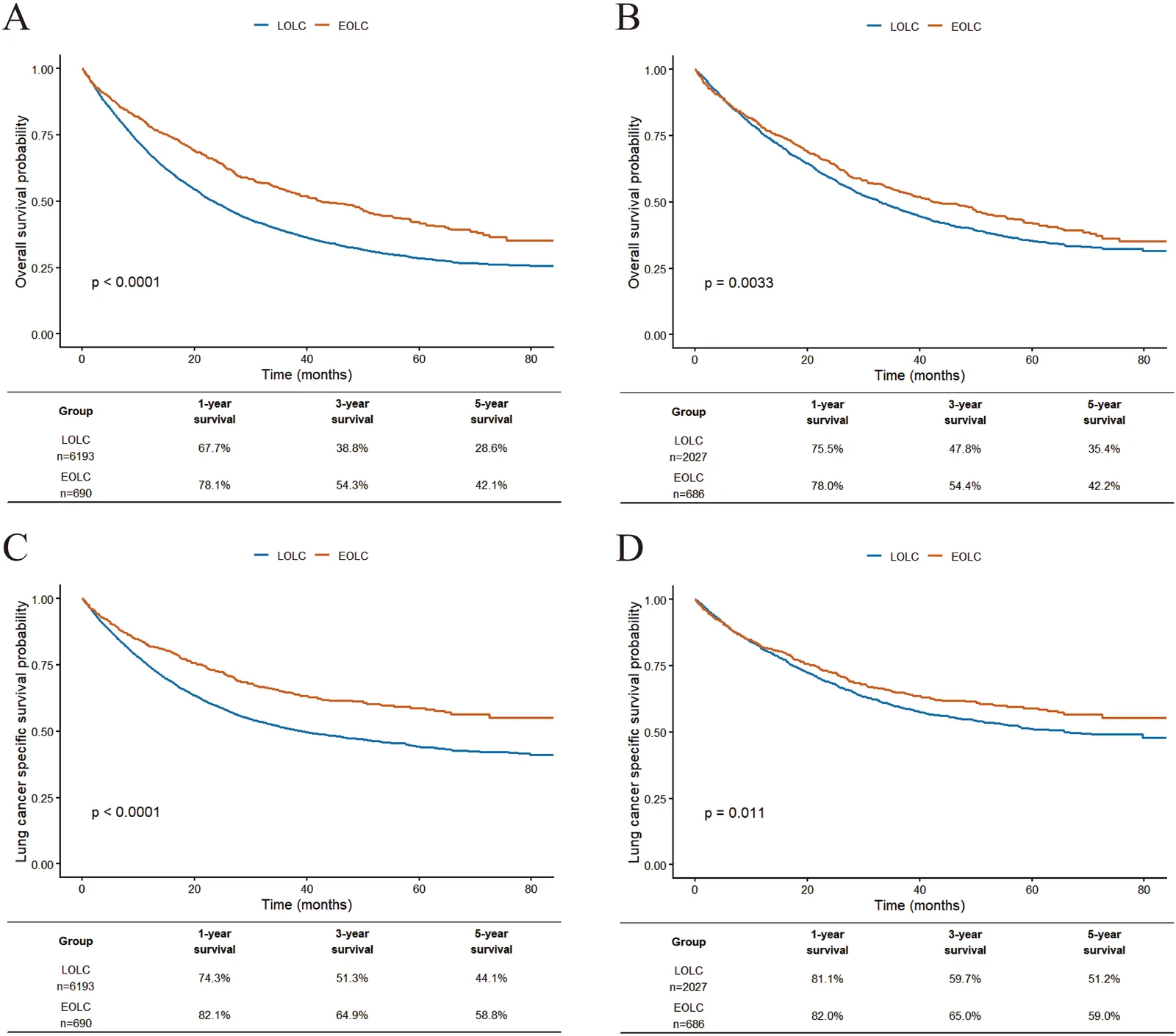
Kaplan-Meier curves for overall survival and lung cancer-specific survival before and after propensity score matching. **(A)** Overall survival before propensity score matching. **(B)** Overall survival after propensity score matching. **(C)** Lung cancer-specific survival before propensity score matching. **(D)** Lung cancer-specific survival after propensity score matching. EOLC, early-onset lung cancer; LOLC, late-onset lung cancer; PSM, propensity score matching; OS, overall survival; CSS, lung cancer-specific survival.

Before PSM, the median OS was 24.8 months for the overall cohort, 42.5 months for the EOLC group, and 23.3 months for the LOLC group. The 1-, 3-, and 5-year survival rates were 78.1%, 54.3%, and 42.1%, respectively, compared with 67.7%, 38.8%, and 28.6% in the LOLC group. Kaplan-Meier survival analysis revealed significant differences in both OS and CSS between the EOLC and LOLC groups, the EOLC group demonstrated superior OS and CSS compared with the LOLC group (P < 0.001; **Figures 2A, C**). After PSM, the median OS was 35.1 months for the overall cohort, 42.7 months for the EOLC group, and 32.9 months for the LOLC group. The 1-, 3-, and 5-year survival rates were 78.0%, 54.4%, and 42.2%, respectively, compared with 75.5%, 47.8%, and 35.4% in the LOLC group. Consistently, the EOLC group demonstrated a better OS (P = 0.003; **Figure 2B**) and CSS (P = 0.011; **Figure 2D**).

Onset type (EOLC vs. LOLC) was modeled as the primary exposure in Cox proportional hazards analyses, with EOLC as the reference. Sequential multivariable adjustment was performed to account for potential confounders (**Table 3**), and analyses were replicated in both the before-PSM and after-PSM cohorts to ensure internal consistency. The scaled Schoenfeld residuals for onset type were randomly and evenly distributed over time with no non-linear trends across all fully adjusted models (**Supplementary Material Figure 3**), confirming that the proportional hazards assumption was well-satisfied for the analyses. Across analytic approaches, LOLC was consistently associated with inferior survival outcomes. In the matched cohort, univariable analysis demonstrated that LOLC conferred a significantly increased risk of all-cause and lung cancer–specific mortality (OS: HR = 1.187, P = 0.003; CSS: HR = 1.200, P = 0.010). These associations persisted after comprehensive adjustment in the before-PSM cohort (Model 4), with LOLC independently predicting a 31.9% higher risk of all-cause mortality and a 34.8% higher risk of lung cancer-specific mortality (OS: HR = 1.319, P < 0.001; CSS: HR = 1.348, P < 0.001). Collectively, these findings indicate that late-onset is an independent adverse prognosticator for both OS and CSS.

**Table 3:** Association between age at onset (EOLC vs. LOLC) and survival outcomes.

Model	OS before PSM		OS after PSM		CSS before PSM		CSS after PSM	
	HR(95%CI)	P	HR(95%CI)	P	HR(95%CI)	P	HR(95%CI)	P
Unadjusted*	1.485(1.338-1.648)	<0.001	1.187(1.059-1.331)	0.003	1.523(1.341-1.730)	<0.001	1.200(1.043-1.381)	0.010
Model 1^a^	1.389(1.251-1.542)	<0.001	1.194(1.065-1.339)	0.002	1.430(1.258-1.626)	<0.001	1.207(1.049-1.389)	0.009
Model 2^b^	1.391(1.253-1.545)	<0.001	1.205(1.075-1.351)	0.001	1.430(1.257-1.626)	<0.001	1.220(1.060-1.404)	0.006
Model 3^c^	1.306(1.175-1.451)	<0.001	1.185(1.056-1.330)	0.004	1.330(1.169-1.513)	<0.001	1.196(1.038-1.377)	0.013
Model 4^d^	1.319(1.186-1.466)	<0.001	1.181(1.053-1.326)	0.005	1.348(1.184-1.534)	<0.001	1.204(1.046-1.387)	0.010

*: Unadjusted model.

a: Adjusted for sex, marital status, and ethnicity.

b: Adjusted for clinical stage, smoker, and family history based on Model 1.

c: Adjusted for tumor size, tumor site, clinical stage, histology, and treatment based on Model2.

d: Adjusted for pleural invasion, bone metastasis, brain metastasis, and liver metastasis based on Model3.

EOLC, early-onset lung cancer; LOLC, late-onset lung cancer; PSM, propensity score matching; OS, overall survival; CSS, lung cancer-specific survival.

### 3.3 Subgroup survival analysis

**Figure 3:**
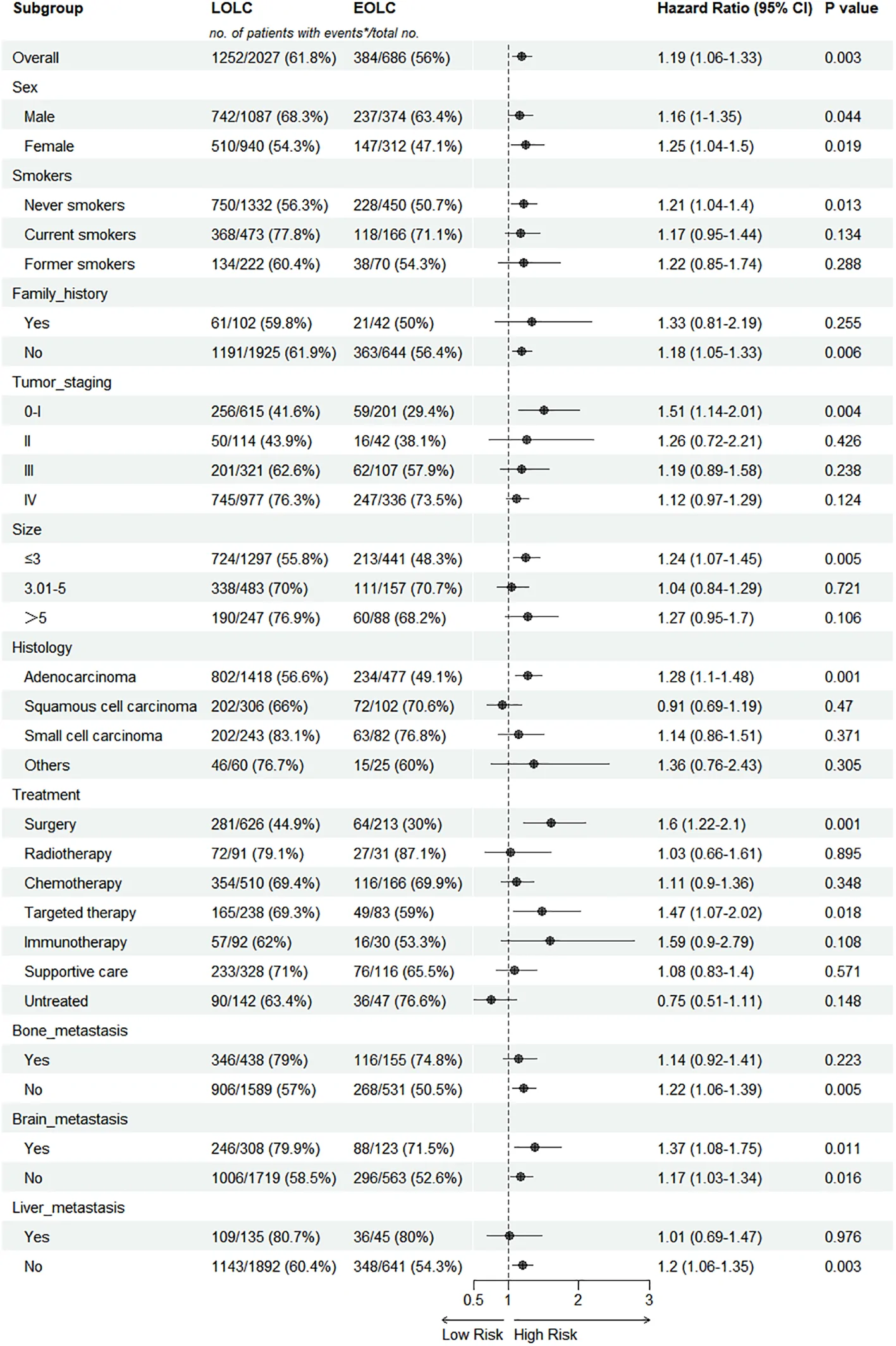
Forest plot of subgroup analyses for overall survival between early-onset and late-onset lung cancer. *: Events were defined as deaths from any cause. EOLC, early-onset lung cancer; LOLC, late-onset lung cancer.

**Figure 4:**
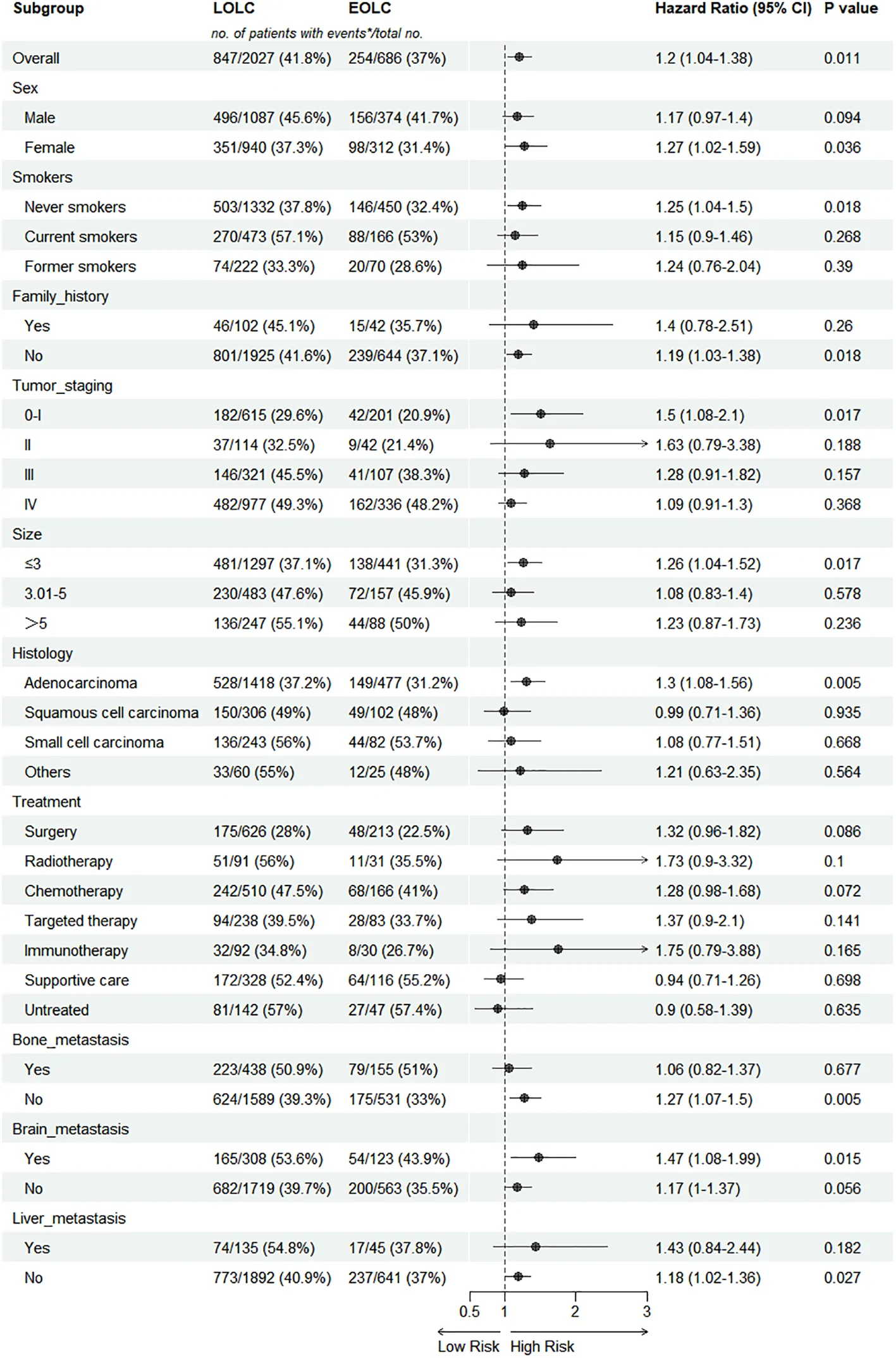
Forest plot of subgroup analyses for lung cancer-specific survival between early-onset and late-onset lung cancer. *: Events were defined as deaths from lung cancer. EOLC, early-onset lung cancer; LOLC, late-onset lung cancer.

Subgroup survival analyses were performed by sex, smokers, family history, tumor staging, tumor size, histology, treatment, and the presence of bone, brain, and liver metastasis (**Figures 3**, **4**). Kaplan–Meier survival curves for tumor staging and treatment analysis are presented in the **Supplementary Material Figures 4–6**. Overall, the survival benefit associated with EOLC was consistently observed across most subgroups. Significant associations were identified among females (HR = 1.25, 95% CI: 1.04–1.50), never smokers (HR = 1.21, 95% CI: 1.04–1.40), patients with tumors ≤3 cm (HR = 1.24, 95% CI: 1.07–1.45), and adenocarcinoma (HR = 1.28, 95% CI: 1.10–1.48). However, no significant survival difference between EOLC and LOLC was observed in stage IV. Subgroup analyses of CSS yielded results generally consistent with those observed for OS (**Figure 4**). However, none of the treatment subgroups demonstrated a statistically significant difference in CSS between EOLC and LOLC. In particular, the survival advantages observed in the surgery and targeted therapy subgroups for OS were no longer evident for CSS (all P > 0.05).

### 3.4 Molecular characteristics

Exploratory molecular analysis was performed in patients with available molecular testing records. Complete molecular testing results were available for 437 patients for EGFR, 312 for ALK, 250 for ROS1, 231 for BRAF, 229 for MET exon 14 skipping, 227 for RET, and 194 patients each for HER2 and KRAS(**Table 4**). Overall, 254 (58.1%) were found to have an EGFR mutation, including 54 (54.5%) with EOLC and 210 (62.1%) with LOLC. However, there was no significant difference between EOLC and LOLC (P = 0.215). HER2 (10.1% vs. 1.6%, P = 0.019) mutations were more frequently observed in EOLC.

**Table 4:** Driver gene mutation status of EOLC and LOLC patients.

Driver genes	Total	EOLC	Mutation rate(n, %)	LOLC	Mutation rate(n, %)	P*
EGFR	437	99	**54(54.5)**	338	**210(62.1)**	0.215
ALK	312	88	**14(15.9)**	224	**27(12.1)**	0.471
ROS1	250	76	**4(5.3)**	174	**2(1.1)**	0.132
BRAF	231	74	**2(2.7)**	157	**1(0.6)**	0.502
RET	227	74	**1(1.4)**	153	**3(2.0)**	1.000
MET	229	73	**1(1.4)**	156	**4(2.6)**	0.927
HER2	194	69	**7(10.1)**	125	**2(1.6)**	0.019
KRAS	194	69	**1(1.4)**	125	**7(5.6)**	0.310

P values were calculated using the Chi-square test or Fisher’s exact test. Bold values indicate mutation rates (n, %) for each driver gene.

EOLC, early-onset lung cancer; LOLC, late-onset lung cancer.

## 4 Discussion

Given the rapid increase in lung cancer incidence among younger adults globally, understanding the characteristics of early-onset disease is now more urgent than ever. Numerous studies have proposed that lung cancer in the young may constitute a unique clinicopathological entity, with differences in sex distribution, stage at diagnosis, pathological features, and prognosis—although published data remain frequently discordant (21–26). Currently, no international consensus has been reached regarding the optimal age cutoff for defining early-onset lung cancer. Previous studies have adopted various cutoffs, including <40 years (27), <45 years (12), and <50 years (28), all of which consistently reported a higher proportion of stage IV disease among younger patients. In contrast, Li et al (29) used a much lower cutoff of <30 years and found that their young patients predominantly presented at early stages, a result that contradicts the aforementioned findings. Adopting stricter cutoffs (<30 years) focuses on very young patients, who account for only a small fraction of all lung cancer cases and often harbor indolent tumors characterized by active immune infiltration, which may be associated with more robust immune surveillance (15). These discrepancies in the definition of EOLC across studies have led to considerable heterogeneity in reported clinicopathological features and survival outcomes. A cutoff set too low may increase the risk of overdiagnosis, whereas a cutoff set too high may introduce bias in the identification of distinct biological characteristics, potentially leading to missed diagnoses. To address this issue, our study employed restricted cubic spline analysis and piecewise Cox regression, and the results support the use of 50 years as the optimal cutoff.

Compared with patients with late-onset lung cancer, our study found that those with early-onset lung cancer had higher proportions of females and non-smokers, which was consistent with previous studies (30–32). This suggests that, apart from traditional risk factors such as smoking, lung cancer in young, non-smoking women may be driven by unique risk factors. One possible reason was that in normal tissues, physiological estrogen facilitated the recruitment and activation of CD8^+^T cells and enhanced the activity of M1-type macrophages-key players in anti-infection and anti-tumor immunity. Conversely, within the tumor microenvironment, physiological or locally produced estrogen might have potentially promoted cell proliferation, causing vascular obstruction and weakening anti-tumor immunity (33). In contrast to previous reports of familial aggregation in early-onset lung cancer, our study showed a comparable distribution of positive family history between the early-onset and late-onset groups (34, 35). This discrepancy may be attributable to the reliance on self-reported family history: some patients might have concealed their family history due to feelings of shame or stigma, resulting in fewer reported positive cases and thus insufficient statistical power to detect a true difference between groups.

Our study also found that patients with EOLC had a higher proportion of advanced-stage (stage IV) disease at diagnosis and were more likely to develop bone and brain metastases, suggestive of an aggressive disease biology. One possible reason for the diagnostic delay was that young patients presented with only limited symptoms and did not believe they were at risk of developing a malignant tumor (36). One study found that 34.6% of EOLC cases were asymptomatic at diagnosis. By the time young patients sought medical care due to symptom onset, their lung cancer might have already developed distant metastases (37). In our study, the percentage of adenocarcinoma in EOLC (69.7%) was significantly higher than that in LOLC. In an Indian study, 85.7% of patients had this subtype (32), and a retrospective study also reported that a high proportion (45%) of patients aged 18–35 years exhibited this feature (10). The reason for the high proportion of adenocarcinoma in young patients remains unclear, but some scholars have suggested that it might be related to the low smoking rate in this population (38). In our study, never-smokers predominantly had adenocarcinoma, whereas current and former smokers had squamous cell carcinoma. Our analysis demonstrated that EOLC was enriched with targetable driver alterations. Interestingly, we identified a relatively higher prevalence of oncogene mutations such as HER2, in the younger cohort, whereas these mutations were rare in older patients. Despite the small sample size for genetic testing, these findings suggest that tumors in young patients with lung cancer may represent a biologically distinct subgroup, and they highlight the importance of urgent molecular analysis to identify targetable mutations for personalized treatment.

Although early-onset lung cancer was more aggressive, in this study, patients with early-onset lung cancer had a much higher median overall survival (42.7 months)than those with late-onset lung cancer(32.9 months). Some studies have shown prognosis to be worse for younger patients, indicating a more aggressive disease (11, 39). Others have shown that there was no significant difference in survival (12, 40). However, it has mostly been demonstrated that younger patients have a better prognosis (9, 28, 41). The main reasons why early-onset lung cancer was more aggressive yet had better survival were as follows. 1)Our study revealed that younger patients were more likely to receive active anti-tumor treatments, particularly surgical resection and chemotherapy, while patients with late-onset lung cancer were more likely to be managed with supportive care alone or no active treatment. Even for stage IV early-onset lung cancer patients, the primary therapeutic approaches were chemotherapy, targeted therapy, and adjuvant surgical intervention. These findings suggest that EOLC patients were more likely to receive curative-intent or intensive anticancer therapy. 2) These younger patients tend to have fewer comorbidities and better tolerance to these treatments, which can be attributed to their more favorable organ function status (42). Therefore, more aggressive multimodality treatments, including surgery, are feasible for young patients with lung cancer and are highly recommended in our daily practice. 3) Age is a well-established risk factor for lung cancer (43). One Mendelian randomization study (44) used phenotypic age acceleration as a validated biomarker of biological aging and demonstrated a causal relationship between accelerated biological aging and increased lung cancer risk. Consistent with these findings, our Cox proportional hazards regression analysis yielded similar results: late-onset (diagnosis age ≥50 years) was an independent adverse prognostic factor, leading to poorer survival in older patients. Therefore, these factors may act together to counterbalance the detrimental effects of delayed diagnosis and advanced stage in EOLC patients, ultimately resulting in a survival benefit for this population. Interestingly, although the incidence of brain metastases was higher in younger patients, younger patients in the brain metastasis group had better survival than older patients. This finding is similar to that reported by Suidan AM (30) and may be attributed to the fact that most young patients had only single-organ metastasis, i.e., “oligometastasis” as proposed by Hellman and Weichselbaum (45).

Current lung cancer screening guidelines are primarily tailored to older adults (≥50 years) with significant smoking histories, thereby excluding a large subset of younger patients who do not meet traditional high-risk criteria. A study in the United States pointed out that the current USPSTF guidelines missed nearly two-thirds of cases, and that lowering the screening age to 40 years could increase the detection rate to 93.9% (46). Similarly, a Chinese study found that the current screening guidelines had a missed diagnosis rate as high as 74.6%, especially among younger individuals and non-smokers (47). This exclusion may contribute to diagnostic delays and worse prognostic outcomes in patients with EOLC. Therefore, it is recommended to refine risk stratification strategies, with a particular focus on women and non-smokers, and to incorporate novel high-efficiency biomarkers to enable early detection. These efforts are crucial for developing targeted prevention strategies and optimizing therapeutic interventions for this underrepresented and often overlooked patient population.

Although this study covered 26 hospitals across different districts in Chongqing, effectively reducing the selection bias commonly associated with single-center studies, it still had certain limitations. Firstly, all data in this study were derived from routine patient care, and the molecular analyses were based on patients with available testing records rather than the entire cohort. Additionally, due to financial constraints, some patients underwent testing for only one or a few targets, resulting in variable sample sizes across individual driver genes. Therefore, the reported mutation frequencies may not be representative of the overall study population and should be regarded as exploratory findings. However, the observations from this study are certainly hypothesis-generating and further highlight the need for more extensive studies to investigate genomic characteristics and to better understand the disease biology of EOLC. Secondly, residual confounding cannot be completely excluded because several important prognostic variables, including performance status, type of surgery, comorbidity burden, pulmonary function, socioeconomic status, and treatment adherence, were unavailable in our registry. Thirdly, lead-time or detection bias cannot be completely ruled out, as differences in diagnostic practices between EOLC and LOLC patients may also have contributed to the observed survival differences. In the future, larger-scale, deeper prospective studies on this topic will be initiated.

In conclusion, EOLC has distinct epidemiology, pathophysiology, and molecular characteristics and survival outcomes compared with LOLC in China. Interestingly, these young patients with lung cancer were predominantly women, non-smokers, and had adenocarcinoma. Although they presented with more advanced stages, greater metastatic burden, and distinct genetic mutations, their survival rate was significantly higher than that of patients with late-onset lung cancer.

## Data Availability

The original contributions presented in the study are included in the article/**Supplementary Material**. Further inquiries can be directed to the corresponding author.
