# Supplementary material for: Clinical characteristics and survival in early-onset lung cancer: a multicenter cohort study
Source: Front Oncol. 2026 Aug 12;16:1906257. doi: 10.3389/fonc.2026.1906257 (PMC13506285; doi:10.3389/fonc.2026.1906257)
Supplement: Supplementary file 1 [file DataSheet1.docx]

**Supplementary Materials**

**Supplementary Appendix 1.** Assessing the validity of using age 50 as a cutoff for early-onset and late-onset lung cancer.

**Supplementary Appendix 2.** After propensity score matching, test the balance of covariates between the early-onset lung cancer and the late-onset lung cancer.

**Supplementary Appendix 3.** Plots of scaled schoenfeld residuals for onset type.

**Supplementary Appendix 4.** Kaplan-Meier curves for overall survival and lung cancer-specific survival stratified by tumor staging and treatment.

**Supplementary Figures**

**Figure 1.** Restricted cubic spline analyses of the associations between age and survival outcomes.

**Figure 2.** Covariant balance before and after propensity score matching.

**Figure 3.** Plots of scaled schoenfeld residuals for onset type.

**Figure 4.** Kaplan-Meier curves for overall survival and lung cancer-specific survival stratified by tumor staging.

**Figure 5.** Kaplan-Meier curves for overall survival stratified by treatment.

**Figure 6.** Kaplan-Meier curves for lung cancer-specific survival stratified by treatment.

**Supplementary Table**

**Table 1**. Piecewise Cox regression analyses of the associations between age and survival outcomes.

**Supplementary Appendix 1**

Using 50 years as the cutoff, restricted cubic spline (RCS) analysis was performed to assess the association between age and survival risk (Figure 1), followed by piecewise Cox regression (Table 1). The RCS curve suggested a nonlinear relationship between age and both overall survival (OS) and cancer-specific survival (CSS). In piecewise Cox regression analyses, age was not significantly associated with survival risk below 50 years (OS: P = 0.369; CSS: P = 0.842), whereas a significant increase in risk was observed above 50 years (OS: HR = 1.026, P < 0.001; CSS: HR = 1.033, P < 0.001). The regression coefficients differed significantly between Model 2a and Model 2b (P < 0.05). Likelihood ratio testing indicated that the piecewise Cox model provided a better fit than the conventional Cox model (P < 0.05). These findings support 50 years as a reasonable cutoff for defining early- and late-onset lung cancer.

Figure 1 Restricted cubic spline(RCS) analyses of the associations between age and OS (A) and CSS (B)

Table 1 Piecewise Cox regression analyses of the associations between age and survival outcomes

|  | | OS | | CSS | |
| --- | --- | --- | --- | --- | --- |
|  |  | HR(95%CI) | P-value | HR(95%CI) | P-value |
| Model1 | | 1.026(1.020-1.028) | <0.001 | 1.030(1.025-1.034) | <0.001 |
| Model2 | Model2a | 1.007(0.992-1.022) | 0.369 | 1.002(0.984-1.020) | 0.842 |
|  | Model2b | 1.026(1.022-1.031) | <0.001 | 1.033(1.028-1.039) | <0.001 |
| Difference of effect between Model2a and Model2b | | 1.020(1.000-1.040) | 0.024 | 1.030(1.010-1.050) | 0.003 |
| Log-likelihood ratio test in Model 1 and Model 2 | |  | 0.027 |  | 0.004 |

Model1:Cox regression model

Model2:Piecewise Cox regression model

Model2a:Piecewise Cox regression model(Age < 50 years)

Model2b:Piecewise Cox regression model(Age ≥ 50 years)

Note: Hazard ratios represent the risk associated with each 1-year increase in age.

Abbreviations: OS, overall survival; CSS, lung cancer-specific survival.

**Supplementary Appendix 2**

After matching, the balance of covariates was assessed (Figure 2, love plot). The results showed that the standardized mean differences (SMDs) for all covariates were <0.1, indicating that the covariates were well balanced between groups after matching.

Figure 2 Covariate balance before and after propensity score matching

**Supplementary Appendix 3**

Figure 3 Plots of scaled schoenfeld residuals for onset type.

(A) Overall survival before PSM;

(B) Overall survival after PSM;

(C) Lung cancer-specific survival before PSM;

(D) Lung cancer-specific survival after PSM.

Abbreviations: PSM, propensity score matching.

**Supplementary Appendix 4**

Figure 4 Kaplan-Meier curves for overall survival and lung cancer-specific survival stratified by tumor staging

(A) Overall survival in stage Ⅰ disease;

(B) Lung cancer-specific survival in stage Ⅰ disease;

(C) Overall survival in stage Ⅱ disease;

(D) Lung cancer-specific survival in stage Ⅱ disease;

(E) Overall survival in stage Ⅲ disease;

(F) Lung cancer-specific survival in stage Ⅲ disease;

(G) Overall survival in stage Ⅳ disease;

(H) Lung cancer-specific survival in stage Ⅳ disease.

Abbreviations: EOLC, early-onset lung cancer; LOLC, late-onset lung cancer.

Figure 5 Kaplan-Meier curves for overall survival stratified by treatment

(A) Overall survival in surgery;

(B) Overall survival in radiotherapy;

(C) Overall survival in chemotherapy;

(D) Overall survival in targeted therapy;

(E) Overall survival in immunotherapy.

Abbreviations: EOLC, early-onset lung cancer; LOLC, late-onset lung cancer.

Figure 6 Kaplan-Meier curves for lung cancer-specific survival stratified by treatment

(A) Lung cancer-specific survival in surgery;

(B) Lung cancer-specific survival in radiotherapy;

(C) Lung cancer-specific survival in chemotherapy;

(D) Lung cancer-specific survival in targeted therapy;

(E) Lung cancer-specific survival in immunotherapy.

Abbreviations: EOLC, early-onset lung cancer; LOLC, late-onset lung cancer.
